# Geometric ordering in bacterial communities

**DOI:** 10.1073/pnas.2526643123

**Published:** 2026-05-12

**Authors:** Melika Gorgi, Summer J. Kasallis, Calvin Trinh, Lizett Ortiz de Ora, Travis J. Wiles, Albert Siryaporn

**Affiliations:** ^a^ Center for Complex Biological Systems, University of California Irvine, Irvine, CA 92697; ^b^ Department of Physics and Astronomy, University of California Irvine, Irvine, CA 92697; ^c^ Department of Microbiology and Molecular Genetics, University of California Irvine, Irvine, CA 92697; ^d^ Department of Molecular Biology and Biochemistry, University of California Irvine, Irvine, CA 92697

**Keywords:** Voronoi tessellations, pellicle formation, bacterial patterning, biofilm formation, entropy

## Abstract

The organization of bacteria has a central role in shaping interactions, dynamics, and composition within communities and microbiomes. Bacteria form distinct spatial patterns that have often been attributed to microbial processes such as chemotaxis, nutrient transport, and signaling. However, common patterns are observed across distinct bacteria and conditions, suggesting that a general organizing principle could direct bacterial organization. Here, we find that the organization of bacteria is explained by geometric ordering that promotes space-filling efficiency, giving rise to geometric patterns known as Voronoi tessellations. We find that the Voronoi Growth Model accurately predicts bacterial pattern formation in diverse conditions including in biofilms at the liquid–air interface, swimming populations, the zebrafish gut, and conditions that promote swarming. The patterns are observed in two and three dimensions, at the cm and mm length scales, across diverse species (*Vibrio cholerae*, *Pseudomonas aeruginosa*, *Escherichia coli*), arise solely from the principles of Voronoi tessellation, and require no detailed knowledge of microbial processes. Entropic considerations show that bacteria provide little or no information about the pattern formation, which is determined solely by their initial positions and environmental conditions. These findings demonstrate that bacterial communities achieve robust, reproducible organization through a universal geometric principle, linking microbial patterning to the broader biological context of multicellular organization.

## Significance

The general principles responsible for bacterial organization at the collective level are unclear. This work shows that large-scale pattern formation among diverse bacteria is explained by geometric ordering, which is also observed in multicellular organisms. This finding suggests that bacteria and multicellular organisms share a common organizational principle and provides insight into the design principles of microbial consortia.

Bacterial communities exhibit spatial organization that significantly influences their interactions, function, and composition (1–3). Understanding the principles of organization is thus important for predicting communal outcomes. Bacterial populations produce remarkably organized and diverse patterns at the collective level including plumes in surface-attached populations within biofilms, “broccoli-like” aggregates in jammed environments, sectors and Turing structures on porous surfaces, pod-like assemblies, and intestinal crypts in the gut, and dendritic patterns in swarms (4–16). Current models that explain microbial organization invoke detailed microbial processes such as chemotaxis, nutrient transport and diffusion, spatial signaling gradients, and quorum sensing (17–29). Such models require detailed knowledge of system dynamics and physical parameters. While some organizational patterns may be species-specific, common pattern formations can emerge across microbial species that are evolutionarily distant (30–36). The emergence of common patterns across distinct species raises the possibility that species-agnostic general principles could underlie some forms of microbial organization.

In multicellular organisms, pattern formation is achieved through directed developmental mechanisms including those that establish a positional coordinate system within the organism or that establish Turing patterns through reaction–diffusion mechanisms (37–40). However, some pattern formations such as the ordering of cells in *Drosophila* eyes, and patterns in tissue and epithelial layers are explained by geometric ordering (41–45). The appearance of geometrically ordered patterns in diverse systems suggests that their formation could be guided by physical constraints and properties of the system rather than on developmental programming provided by cellular processes.

We consider whether geometric ordering alone can predict the organization of bacterial communities. Microbial collectives and communities, which contain different bacterial species, must organize according to principles that govern space filling. Tessellations are space-filling patterns that arise through geometric rulesets. Voronoi tessellations (also known as a Dirichlet tessellations) have been applied extensively in the realm of computational geometry to optimize resource utilization to solve a wide range of issues in epidemiology, communications, and resource management, which relate to space allocation, coverage, and efficiency. The organization of epithelial cells in multicellular organisms in particular is described by Voronoi tessellations (43, 46, 47). Given that bacteria experience persistent competition for resources and space in natural environments, it is possible that their spatial structure could follow a similar organizing principle.

We investigated the spatial organization of diverse bacteria including *Escherichia coli*, *Vibrio cholerae*, and *Pseudomonas aeruginosa* under diverse conditions including agar surfaces, liquids, and in the zebrafish gut. The latter two species are pathogens that cause severe infections including acute diarrheal disease, lung and skin infections, and sepsis (48, 49). Understanding how these pathogens organize at the collective level could provide insight into their pathogenesis. We found that the growth of these bacterial populations mirrors a method for constructing Voronoi tessellations and that these patterns arise solely from radial growth and collision-induced inhibition. Our findings demonstrate that details of microbial processes are dispensable for these pattern formations and that the fundamental outcome of bacterial growth is to fill all available space according to a geometric ruleset.

## Results

### Swimming *V. cholerae* Populations and *P. aeruginosa* Form Voronoi Tessellations.

**Fig. 1.**
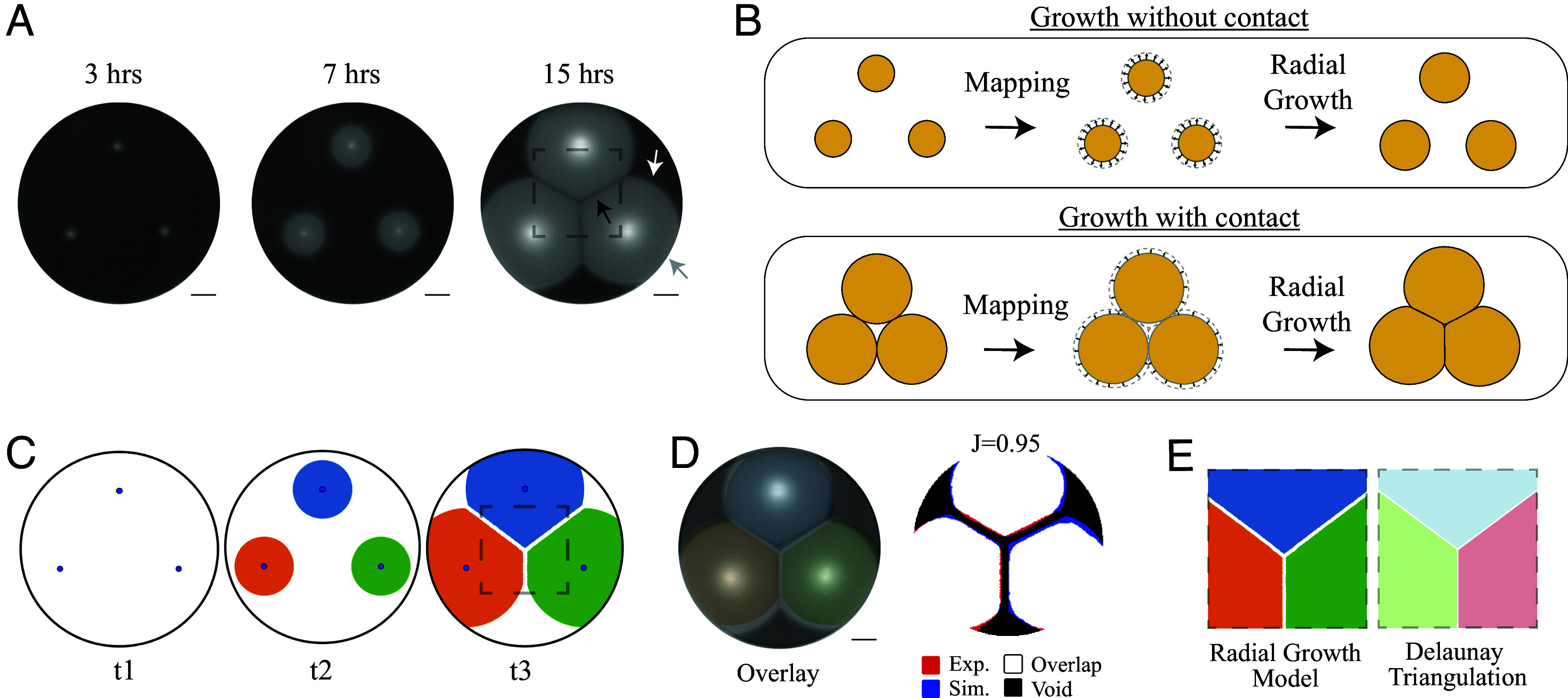
Swimming bacteria form Voronoi tessellations. (*A*) Timelapse of *V. cholerae* (ZWU0020) populations that were inoculated in three positions 4 cm apart onto 0.2% agar, shown at 3, 7, and 15 h. A linear low-density boundary appears at the interface where populations collide (black arrow). Expansion is also arrested by the plate wall, producing a curved boundary (gray arrow). Populations that have not collided continue to expand radially (white arrow). The dashed box indicates the region containing the Voronoi tessellation. (Scale bar, 1 cm.) (*B*) Schematic of the Radial Growth Model (RGM). Populations expand radially with circular symmetry when there is no boundary contact; growth is inhibited at boundary contact points, generating local linear interfaces. (*C*) Simulation of the RGM using the initial seeding configuration in *A*, showing snapshots at three time points: initial seeding (t1), intermediate radial expansion (t2), and following boundary collision (t3). The dashed box indicates the collision region of the RGM. (*D*) Overlay of the experimental 15-h image and t3 of the simulation (*Left*) and comparison of image masks indicating overlap/agreement (white), where only the experiment (red) or simulation (blue) are observed, or where neither is observed (black). The Jaccard image similarity index (J) is given. (*E*) Boundaries created by the RGM (*Left*) and a Voronoi diagram produced by Delaunay triangulation (*Right*).

We tracked the growth of *V. cholerae* on agar surfaces that promote flagellar-mediated swimming motility. Strains were inoculated at a single point and swam outward in all directions, resulting in the homogeneous radial expansion of the bacteria on the surface. To understand how multiple bacterial populations organize in tandem, we spotted three inoculums of the same strain in a triangular arrangement with 4 cm separations (Fig. 1*A*, 3 h). Individual populations expanded radially at each inoculum point (Fig. 1*A*, 7 h, and Movie S1). When the outer boundary of a swimming population collided with a different population, both stopped advancing at the collision point, creating a low cell density linear boundary between the two populations (Fig. 1*A*, 15 h, black arrow). The populations did not appear to mix significantly after they collided, suggesting that the collision of different populations limited their motility. Parts of the population that had not collided retained circular boundaries and continued to advance radially (white arrow). The portion of the population that collided with the petri dish wall was also expansion-inhibited and took on the shape of the wall (gray arrow).

To understand how this organization arises in *V. cholerae*, we performed a computational simulation of their growth. To replicate the observed *V. cholerae* growth features, we constructed a radial growth model (RGM) using the rule that bacterial populations expand radially but are inhibited at points where the boundary contacts another population (Fig. 1*B*) or simulation boundary. Details of microbial processes such as chemotaxis or nutrient availability were not incorporated in order to assess whether a geometric rule was sufficient to replicate the growth features. Using the triangular arrangement in our *V. cholerae* experiments (Fig. 1*C*, t1) as the initial condition, we found that this parameter-free model recapitulated *V. cholerae* population expansion, exhibiting expanding circular disks at intermediate times (Fig. 1*C*, t2 and Movie S1). At later times, the RGM replicated distinct features observed in the *V. cholerae* data, including linear boundaries at the collision boundaries, circular boundaries where populations continued to expand, and collision with the petri dish boundary (Fig. 1*C*, t3 and Movie S1). The similarity between the RGM simulation and experimental image was visually evident in an image overlay, which yielded a Jaccard image similarity index value of 0.95 (Fig. 1*D*), for which the maximum value of 1 indicates identical images. Geometric similarity was also assessed using Chamfer distance (50), for which a smaller value indicates greater similarity, yielding a value of 0.67 mm (*SI Appendix*, Table S1). For reference, a visually dissimilar geometry (*SI Appendix*, Fig. S1*A*) yielded a significantly higher Chamfer distance of 6.41 mm.

The RGM is an implementation of the Voronoi Growth Model, a mathematical ruleset that generates geometric maps known as Voronoi tessellations or diagrams (51, 52) that efficiently divide space between separate entities. The Voronoi Growth Model has five requirements: (i) initial positions of entities are present at the same time, (ii) initial positions do not change, (iii) growth commences immediately in all directions from the initial position, (iv) growth rate is the same for all entities, and (v) growth ceases upon contact with another entity (51). Indeed, *V. cholerae* growth and RGM fulfills all of these requirements. The direct correspondence between the Voronoi Growth Model, RGM, and *V. cholerae* growth suggests that the communal organization of *V. cholerae* could be Voronoi tessellations.

Voronoi tessellations can also be formed through alternative algorithms including Delaunay triangulation. This algorithm connects points whose domains share a boundary and is widely used for fast computational partitioning (52–55). In contrast to the Voronoi Growth Model, Delaunay triangulation does not involve growth but instead performs planar triangulation between initial points, forming linear boundaries. Due to its disparate approach, the algorithm cannot capture radial expansion, intermediate growth phases, or boundaries where the *V. cholerae* did not collide (areas outside of the dashed box in Fig. 1 *A* and *C*). Despite the substantial differences between the RGM and Delaunay triangulation, the *V. cholerae* collision boundaries (dashed box in Fig. 1*C*) were identical to the Voronoi diagrams formed by Delaunay triangulations (Fig. 1*E*). These results support the interpretation that the boundaries formed by *V. cholerae* collisions are Voronoi tessellations. In addition to reproducing the Voronoi tessellations formed by colliding populations, the RGM accurately predicted collision-free boundaries and replicated aspects of *V. cholerae* expansion dynamics (Movie S1). In contrast, the Delaunay triangulation replicates only the linear boundary that arises between *V. cholerae* populations that have collided.

**Fig. 2.**
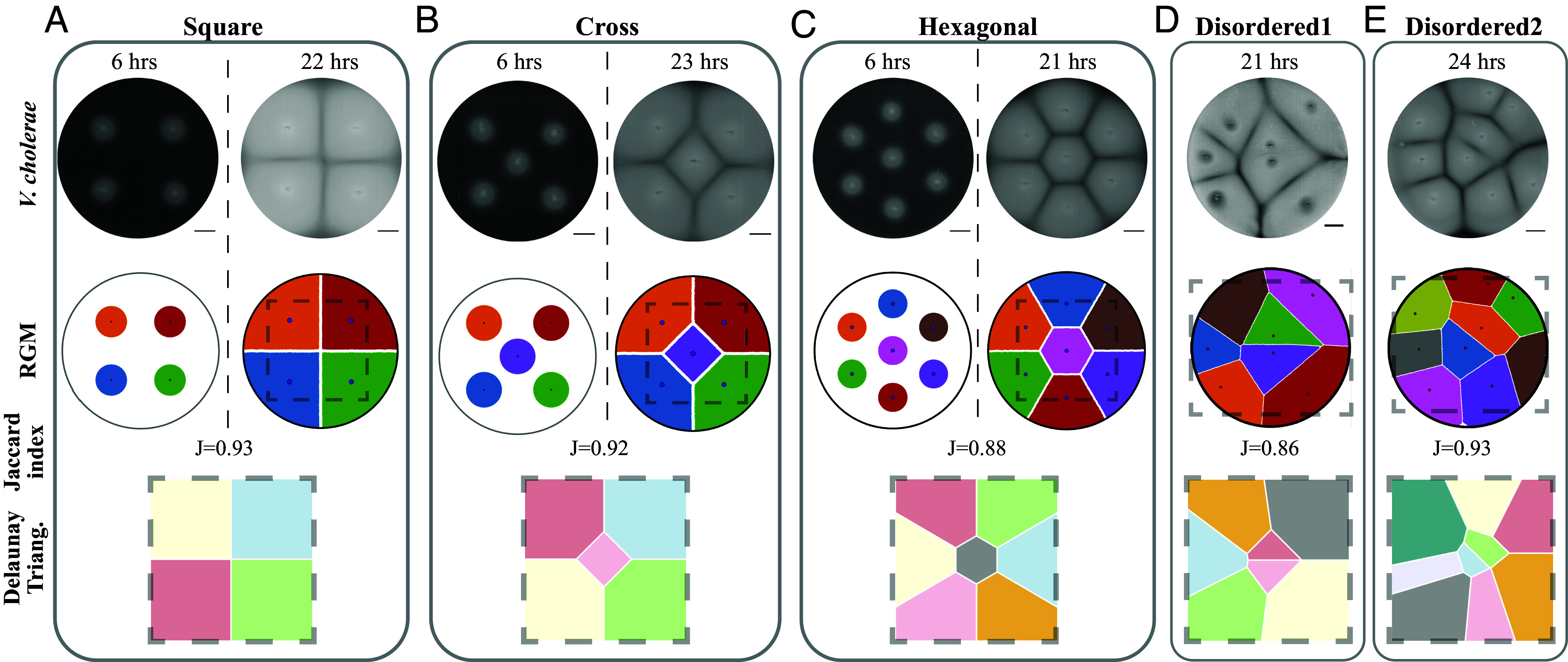
Voronoi tessellations emerge across diverse initial arrangements. (*A*–*C*) Images of *V. cholerae* (ZWU0020) that were initially inoculated with square, cross, and hexagonal arrangements with 3 cm separations on 0.2% agar. First row: images at an early and a late time point (6 and 21 to 23 h). Second row: simulations using the RGM and the same seeding arrangements. Third row: The Jaccard image similarity index (J) is reported. Fourth row: Voronoi diagrams produced through Delaunay triangulations computed from the same seeding arrangements. (*D* and *E*) Images of *V. cholerae*, RGM simulations, Jaccard index, and Voronoi diagrams for bacteria that were initially seeded in disordered arrangements. Dashed boxes in the RGMs indicate the area containing the Voronoi tessellation. (Scale bar, 1 cm.)

To further investigate the ability of the RGM to predict *V. cholerae* organization, we inoculated *V. cholerae* in increasingly complex initial arrangements: square, cross, and hexagonal (Fig. 2 *A*–*C*). Consistent with the triangular arrangements, colonies produced uniform space-filling patterns containing linear collision boundaries and overall patterns that were uniform and symmetric. The RGM replicated the radial boundaries of *V. cholerae* expansion at intermediate times as well as the emergence of linear collision boundaries at later times. Inoculation using irregular positions produced disordered space-filling patterns (Fig. 2 *D* and *E*) that were also identical to the RGM-generated patterns. These configurations yielded Jaccard indices between 0.86 to 0.93, indicating significant image similarity (*SI Appendix*, Table S1 and Fig. S1*B*). For all arrangements, the collision boundaries produced by the RGM were identical to the Voronoi tessellations produced by Delaunay triangulation, supporting the interpretation that collision boundaries are Voronoi tessellations.

**Fig. 3.**
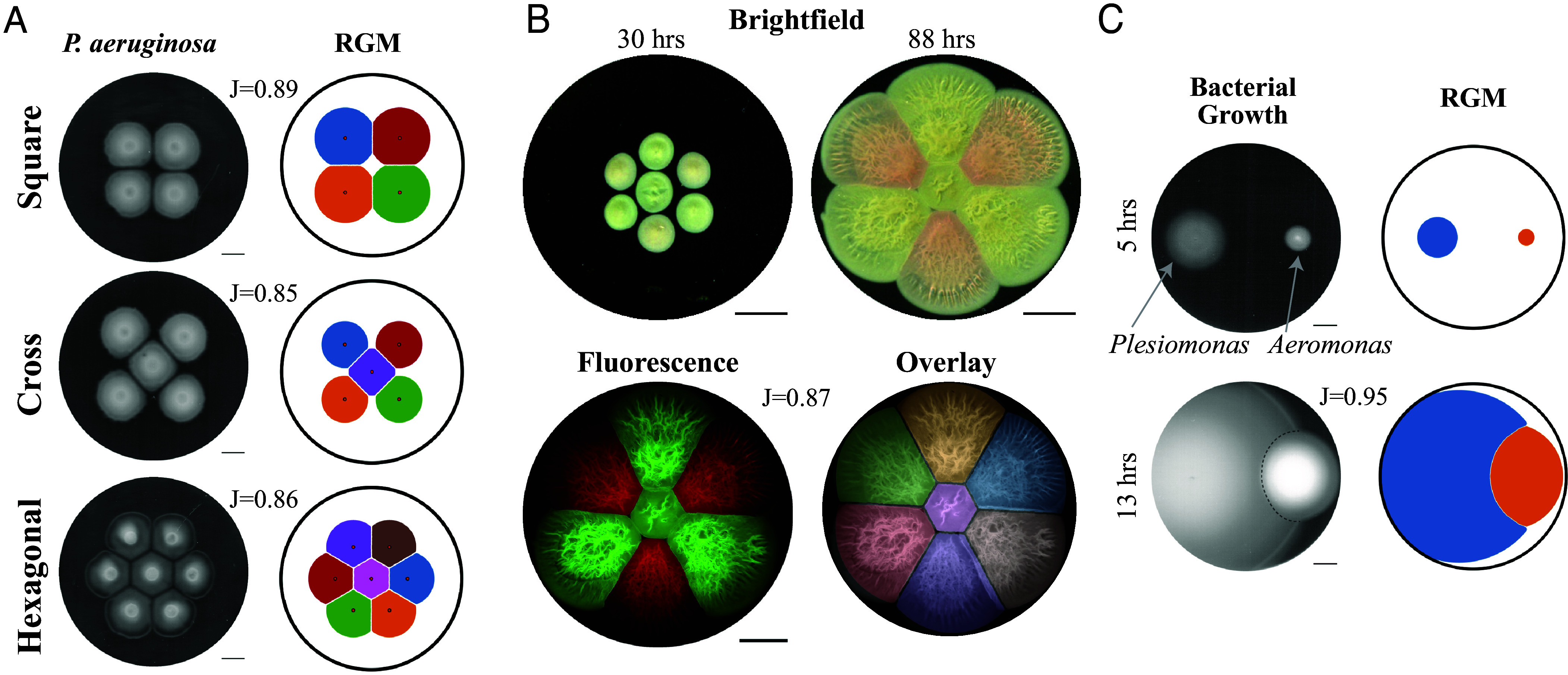
Voronoi tessellations emerge across different species, in a biofilm, and within mixed-species communities. (*A*) Images of *P. aeruginosa* on 0.5% agar, which promotes swarming, and corresponding RGM simulations of bacteria at 14, 14.5, and 9.5 h following initial inoculations in square, cross, and hexagonal arrangements, respectively. (*B*) *V. cholerae* (TW402 and TW421) inoculated in a hexagonal configuration spaced 5.5 mm apart on media containing 0.05% methyl cellulose and 0.2% hydroxyethyl agarose, at indicated incubation times (*Top*). Fluorescence image at 88 h (*Bottom*, *Left*) and the same image in grayscale overlaid with a hexagonal RGM simulation (*Bottom, Right*). (*C*) Images of *Plesiomonas* (faster growth) and *Aeromonas* (slower growth) at 5 and 13 h following inoculation on 0.2% agar and corresponding RGM simulations. J values indicate Jaccard indices. (Scale bar, 1 cm.)

Since the RGM is parameter-free, we expected that the patterns should be observable across diverse bacteria and conditions. Indeed, inoculation of *P. aeruginosa* in uniform square, cross, and hexagonal arrangements on media that promotes swarming produced space-filling patterns identical to the RGM (Fig. 3*A*). At later times, dendritic patterns associated with swarming were observed at boundaries that continued expansion but the Voronoi tessellations at collided boundaries remained unchanged (*SI Appendix*, Fig. S1*C*).

### Biofilms Form Voronoi Tessellations.

Given the robust formation of Voronoi patterns across different species and that the requirements of Voronoi growth are fulfilled simply by radial growth and contact inhibition, we hypothesized that the tessellations arise in biofilms. We inoculated flagellum-defective *V. cholerae* mutants to promote biofilm formation (56) in a compact (6 mm from center) hexagonal configuration. Experiments were performed on the surface of liquid medium containing 0.05% methylcellulose and 0.2% hydroxyethyl agarose, which together mimic the viscoelastic and shear-thinning properties of mucus in host environments (57–59). Populations expanded radially at the air–liquid interface in the form of pellicles (Fig. 3*B*, *SI Appendix*, Fig. S1*D*, and Movie S2). Boundary expansion was inhibited upon contact with neighboring populations, transforming radially symmetric populations into a hexagonal Voronoi tessellation (Fig. 3*B*, *SI Appendix*, Fig. S1 *D* and *E*, and Movie S2). The pattern yielded a Jaccard index of 0.87 with the RGM simulation (additional metrics in *SI Appendix*, Table S1) and was identical to the hexagonal pattern observed with swimming *V. cholerae* on semisolid agar surfaces (Fig. 2*C*). Additionally, wrinkles appeared in the biofilm structure concomitant with contact with neighboring populations (Fig. 3*B*, *SI Appendix*, Fig. S1*D*, and Movie S2), which we attribute to compressive forces imparted by the neighboring populations (60). These results demonstrate that Voronoi tessellations arise in a *V. cholerae* biofilm, without a motility mechanism, at the liquid–air interface in viscoelastic media, and across multiple length scales.

### Multispecies Populations Form Modified Voronoi Tessellations.

In microbial communities, different bacterial species have distinct growth rates (61), which could affect communal organization. We assessed the impact of growth rate on community organization using *Plesiomonas sp.* and *Aeromonas* *sp.*, which also colonize the zebrafish gut (62). Using the same growth conditions as the *V. cholerae* swim assays, populations of both species expanded radially, with *Plesiomonas* expanding at a higher rate than *Aeromonas* (Fig. 3*C*, 5 h and Movie S3). *Plesiomonas* partially engulfed *Aeromonas*, with their interaction producing two notable features: a curvilinear boundary between the two species and an oval-shaped *Aeromonas* population (Fig. 3*C*, 13 h and Movie S3). Incorporation of distinct growth rates into the RGM replicated both of these features (Fig. 3*C*, *SI Appendix*, Fig. S1*F*, and Movie S3), which are consistent with weighted Voronoi tessellations (51). These results support the interpretation that bacterial populations that have distinct growth rates form Voronoi tessellations.

**Fig. 4.**
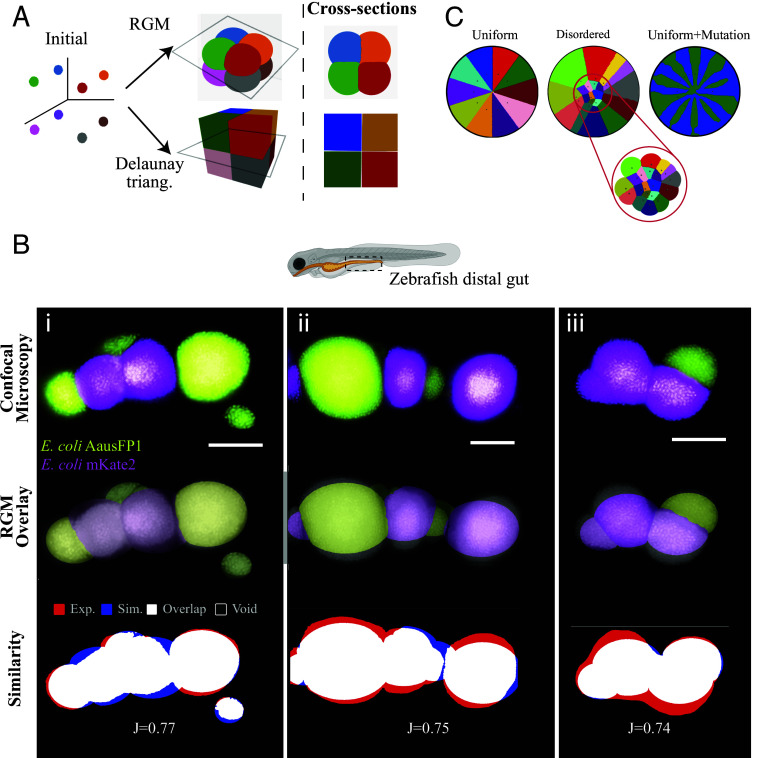
Gut colonization organization is consistent with Voronoi tessellation in 3D. (*A*) Three-dimensional extension of the RGM, with comparison to Delaunay triangulation. Seeds placed at the vertices of a cube generate expanding spheres. Planar cross-sections produce the same type of partitions as in 2D, consistent with a 3D Voronoi tessellation. (*B*) Confocal images of the Zebrafish distal gut colonized by two otherwise identical fluorescent *E. coli* strains (AausFP1, green; mKate2, magenta). Credit line: Confocal images (*Top*) show segregated populations separated by linear or curved interfaces. Populations were reconstructed using the RGM (*Middle*) and estimated seeding centers. Comparison between confocal images and RGM simulations (*Bottom*) indicating overlap/agreement (white), where only the experiment (red) or simulation (blue) is observed, or where neither is observed (black). (Scale bar, 15 µm.) (*C*) Initial seeding using the RGM using uniform seeding positions produces uniform sectors (Uniform); disordered seeding produces heterogeneous sector sizes and polygonal partitions (Disordered); introducing a variation in growth rate with uniform seeding generates pronounced radial spokes and sector dominance (Uniform + Mutation). Sections of panel B were reproduced from ref. 13, which is licensed under CC BY 4.0.

Bacteria grow along three dimensions in natural and host environments, whereas our *V. cholerae* analysis and RGM have been predominantly constrained to two dimensions. *V. cholerae* growth on agar surfaces is not strictly two-dimensional because the population expands vertically on petri dishes, but this thickness is negligible compared to their radial expansion distance. We sought to analyze communal organization in three dimensions and therefore incorporated expansion along an additional axis into the RGM. To test the 3D RGM, bacteria were initially arranged at vertices of a cube. The RGM produced expanding spheres that gave rise to boundaries that were identical to those formed by 3D Voronoi tessellations established through Delaunay triangulation (Fig. 4*A*).

We hypothesized that the 3D RGM could replicate the organization of bacteria in a host environment, using the distal gut of zebrafish as a model. Zebrafish were previously inoculated with *E. coli* that expressed AausFP1 and mKate2 fluorescent proteins and had swimming motility intact (data from ref. 13). Strains were inoculated in 4-d-old germ-free larvae, as described in the *Materials and Methods* section. A dense mucus lining is present in the gut, which contributes to limiting bacterial motility and promoting the development of cohesive bacterial aggregates (63, 64). Growth within this viscoelastic environment could facilitate expansion that is clonal and radial, which aligns with the properties of the RGM.

*E. coli* formed segregated populations that were separated by linear or curvilinear boundaries (Fig. 4*B*). Using the approximate centers of the populations as the initial seeding positions and empirically determining relative growth rates, the RGM replicated distinct features of the gut *E. coli* including segregated strain populations, linear and curvilinear boundaries, and diverse population patterns including those that were spherical, hemispherical, oval, and irregular-shaped in 3D (Fig. 4*B* and *SI Appendix*, Fig. S2). The *E. coli* patterns and RGM simulations had Jaccard indices (Fig. 4*B* and *SI Appendix*, Table S1) that were slightly lower but comparable to those observed in *V. cholerae* (Figs. 1, 2, and 3*B*). These results suggest that the organization of *E. coli* in the zebrafish gut can be approximated by Voronoi tessellations that are produced through the RGM.

### Capturing Colony Growth Using the RGM.

We considered the potential for the RGM to explain bacterial organization on firm (1.5 to 2%) agar surfaces that are used ubiquitously. Here, bacteria expand radially in the form of colonies. Their expansion is inhibited by the crowding of neighboring bacteria, producing segregated subpopulations that have sectored organization (4, 5). Using an initial arrangement in which distinct points were uniformly distributed about an annulus, the RGM produced segregated sectors that were uniform in size (Fig. 4*C*). Disordered arrangements that mimic random inoculum positions produced sectors containing diverse sizes and shapes (Fig. 4*C*). By allowing for the possibility of different growth rates due to mutation, diverse communal organization partitions and boundaries were observed (Fig. 4*C*), similar to those observed on petri dishes previously (4, 5). These findings highlight the versatility of the RGM and suggest that colony growth could be modeled as Voronoi tessellations.

## Discussion

Understanding the principles of collective cellular organization and pattern formation in bacteria has been a longstanding goal. Here, we have found that the organization of bacterial populations can be described by an RGM, which is an implementation of the Voronoi Growth Model (51) ruleset. This result has significant implications for bacterial pattern formation, information encoded in bacterial organization, and the relationship between pattern formation in bacterial populations and multicellular organisms, which are discussed below. The finding has broad applications, as microbial communities can be designed to precise organizational requirements using this geometric principle, an overarching goal of engineering living materials (65).

### Requirements for Voronoi Tessellation.

Diverse patterns of bacteria have been modeled using biophysical details of bacterial physiology and cellular processes (i.e., motility, signaling). Our findings suggest that bacterial organization at collision boundaries between populations can be described using geometric ordering in the form of Voronoi tessellations. In addition, the RGM accurately predicts boundaries that have not collided. Voronoi diagrams have been used to model territory to predict bacterial colony size (34). Our results show that the spatial architecture of a bacterial community itself constitutes a Voronoi tessellation.

The requirements for the formation of bacterial Voronoi tessellations are that: (i) clonal populations must expand radially and (ii) growth expansion must be arrested upon contact with the boundary of another population. Our data demonstrate that diverse conditions fulfill these criteria, including swimming populations of *V. cholerae*, *V. cholerae* biofilms formed on surfaces of a viscoelastic medium, *P. aeruginosa* on agar, and *E. coli* in the zebrafish gut, representing both two- and three-dimensional environments across mm and cm length scales. Importantly, the result demonstrates that detailed biophysical parameters are dispensable for predicting the large-scale organization of bacteria in our experimental conditions. These conditions share a common aspect: expansion is driven by a process such as swimming, high-density growth in biofilms, or swarming, and is opposed by mechanical resistance that could arise from viscoelasticity or surface tension from the environment. Voronoi tessellations form because bacteria overcome the environmental resistance, but do not overcome the resistance created by contact with a high-density neighboring population.

Conditions that give rise to Voronoi tessellations could be fulfilled a number of ways in microbial environments including the presence of another microbial species, mechanisms that inhibit growth upon contact (66), barriers that cause physical exclusion, and boundaries created by bacterial products such as surfactants (67) or signaling molecules (16). The observation of similar organization in other bacteria and fungi (30–36) suggests that the Voronoi pattern formation is robust, i.e., it is not sensitive to details of a particular strain or the environment. This insensitivity stands in contrast to systems such as chaotic or fractal systems, where minor perturbations in initial conditions produce macroscopic consequences. The observation of Voronoi patterns across strains and conditions raises a fundamental question: what underlies the robustness of their formation? We speculate that the phase space of microbial growth dynamics contains an attractor that directs systems to converge upon the formation of Voronoi tessellations.

Clearly, Voronoi patterns do not appear in all microbial systems. Such patterns are not expected if radial growth or boundary inhibition is perturbed, such as by external mechanical forces, the presence of flow, lack of mechanical resistance, or population mixing. For example, mechanical stress at boundaries creates instabilities that produce patterns such as buckling or wrinkling (68, 69). Conversely, the observation of a Voronoi pattern in a microbial collective would imply the absence of such external factors, that population growth is radial and clonal, and that boundaries exhibit contact inhibition. While the formation of the patterns was not dependent on distance between populations in our experiments, we anticipate that at smaller length scales such as at the single-cell level, Voronoi tessellations are less likely to be observed because expansion geometries are not typically symmetric and are dependent on details associated with cell shape and packing. An additional consideration that is likely to affect Voronoi pattern formation is cell density, which is strongly influenced by growth rates and spatial expansion rates. High spatial expansion rates through processes such as motility coupled with low growth rates could produce low cell densities that would promote population mixing instead of boundary contact inhibition. Future work will need to determine the physical configurations that promote or abolish Voronoi patterns.

### Entropic Changes and Relationship to Multicellularity.

The observation of Voronoi tessellations in bacteria raises fundamental questions about entropic changes due to microbial growth. For example, multicellular organisms organize using positional information in which cells establish an axis coordinate (37). Pattern formation in these organisms increases entropy by following the maximum entropy principle (70), which states that systems evolve toward the state that maximizes overall entropy of the system. However, it is unclear whether the creation of Voronoi patterns by bacteria increases entropy. Radial expansion clearly increases the spatial distribution of the population, consistent with the maximum entropy principle increasing spatial entropy. However, the pattern that evolves is determined solely by the initial inoculum positions of the bacterial population. The configurational entropy change due to pattern formation is therefore zero. The pattern formation is thus consistent with the maximum entropy principle but with no configurational entropic change. The zero-entropy result implies that the pattern formation is not dependent on the details of molecular, cellular, or environmental processes that occur during bacteria growth. Rather, the information required for the pattern formation is encoded by the initial position of bacteria.

Voronoi patterns are observed in multicellular organisms, particularly in epithelial layers (41–45). The appearance of these patterns in both microbes and multicellular organisms suggests that these organisms share the same organizing principle. In both cases, the organization emerges when cells or structures expand from fixed centers and are constrained by neighboring domains. Geometric ordering may thus be a universal mechanism of biological organization that could occur across multiple length scales, cell physiologies, and cell types to satisfy particular growth needs. The observation of Voronoi tessellations in bacteria suggest that they fill space and utilize resources efficiently. Other geometric orderings could arise given other specific requirements of cellular collectives.

## Materials and Methods

### Bacterial Strains and Growth Conditions.

*V. cholerae* strain ZWU0020, *Plesiomonas sp.* strain ZOR0011, and *Aeromonas* sp strain ZOR0001 (71) were cultured overnight from a frozen stock at −80 °C in five milliliters of Tryptic Soy Broth (TSB) (BD, Franklin Lakes, NJ) at 30 °C with shaking. *V. cholerae* strains TW402 (64) or TW421 (71), which are Δ*pomAB* motility mutants of ZWU0020 that constitutively express dTomato or sfGFP, respectively, or *P. aeruginosa* strain PAO1F (72) were struck out from a frozen stock onto Luria-Bertani Broth-Miller (LB) (BD, Franklin Lakes, NJ) plates containing 2% (w/v) Bacto agar (BD), and incubated overnight at 37 °C. Single colonies were inoculated into liquid LB broth and incubated for 16 to 18 h in a rolling drum at 18 rpm and 37 °C.

#### 0.2% agar assays (for swimming).

Overnight cultures of ZWU0020, ZOR0011, and ZOR0001 were suspended in 1 mL 0.7% NaCl and inoculated onto plates containing TSB and 0.2% Bacto agar (VWR, Radnor, PA). Plates were incubated at 30 °C and imaged every 20 min for 24 h using an Epson V39 II scanner (Epson, Los Alamitos, CA). Raw images available at (73).

#### 0.5% agar assays (for swarm-promotion).

Five microliters of overnight cultures of PAO1F were inoculated onto LB plates containing 0.5% (w/v) Bacto agar (BD) that were prepared in a laminar flow hood at 300 cubic ft/min at 50% ambient humidity, as described previously (67). Images were acquired every 30 min for 18 to 20 h using an Epson V39 II scanner that was controlled using RoboTask (available at http://robotask.com), and were processed using ImageJ 1.54d (NIH, Bethesda, MD). Raw images available at (73).

#### Methylcellulose-LMP agarose assays (for biofilms).

Liquid medium was prepared by combining LB broth with 2-hydroxyethyl agarose (SeaPlaque, Lonza, Rockland, ME) at 42 °C and 1% 4000 cP methyl cellulose (Sigma-Aldrich M0512, Saint Louis, MO) which was stored at 4 °C, to a final concentration of 0.2% 2-hydroxyethyl agarose and between 0.05 to 0.15% methyl cellulose. Petri dishes containing the liquid media were cooled to room temperature for at least 30 min before inoculation. Overnight cultures of TW402 and TW421 were inoculated onto the liquid surface in petri dishes using 27G 13-mm needles or metallic pins that were arranged in a hexagonal pattern spaced 5.5 or 6 mm apart using a 3D-printed mold. Petri dishes were incubated for 3 to 4 d at 37 °C with darkened lids and imaged at 30-min intervals using an Epson Perfection V370 scanner (Epson, Los Alamitos, CA) that was controlled by RoboTask (robotask.com), and images were processed using ImageJ version 1.54d (NIH, Bethesda, MD). Raw images available at (73).

### Bacterial Colonization of the Zebrafish Gut.

Data for zebrafish were previously acquired (13). Briefly, and imaged every 20 min for wild-type (AB) and transgenic TgBAC (nkx2.2a:megfp) zebrafish embryos were derived germfree and colonized with two *E. coli* HS human gut isolate strains (13) that constitutively express the AausFP1 (green) or mKate2 (magenta) fluorescent protein but are otherwise identical. Fertilized eggs from adult mating pairs were incubated in sterile embryo media (EM) containing ampicillin 100 μg/mL, 10 μg/mL gentamicin, 250 ng/mL amphotericin B, 1 μg/mL tetracycline, and 1 μg/mL chloramphenicol, for ~6 h prior to washing embryos with EM containing 0.1% polyvinylpyrrolidone-iodine, then EM containing 0.003% sodium hypochlorite. Sterilized embryos were transferred into T25 tissue culture flasks containing 15 mL of sterile EM at a density of one embryo/mL and incubated at 28.5 °C. Embryos were sustained on yolk-derived nutrients during experiments. Bacterial strains were grown overnight as described in the *Bacterial* *Strains and Growth Conditions* section above. For each bacterial strain, 1 mL of overnight culture was centrifuged for 2 min at 7,000× g, washed once in sterile EM, and added to individual flasks containing 4-d-old zebrafish larvae, for a final density of 10^6^ bacteria/mL. Images of zebrafish distal guts were acquired by fluorescence microscopy as described in ref. 13.

### Simulations Using the RGM.

All simulations were implemented in Python. We developed two main types of simulation frameworks for modeling bacterial colony expansion in both two and three dimensions. The first framework assumes identical growth rates for all populations, while the second allows for heterogeneous growth rates among populations.

In the 2D model with uniform growth rates, each population is initialized with a defined position on a 2D surface (representing the petri dish) and a constant growth rate is assigned globally. A custom collision function determines when the growth of a population should cease. This function imposes two constraints: 1) a global boundary, typically circular, representing the physical limits of the petri dish; and 2) a local minimum distance condition (dmin) that must be satisfied to permit further expansion. At each time step, all populations are allowed to grow, and their continued expansion is checked using the collision function to prevent overlap.

For simulations involving populations with differing growth rates, the same structure is used, but an array of growth rates is defined and individually assigned to each population at initialization. The collision function and expansion rules remain unchanged, with growth occurring independently according to each population’s assigned rate.

The 3D simulation framework extends the approach to model spherical colony expansion in a three-dimensional space. As in 2D, both uniform and heterogeneous growth scenarios were implemented, with spherical boundaries replacing circular ones and 3D distance calculations used in the collision detection step. The raw code used for simulations is available at (74).

### Voronoi Diagrams.

To visualize Voronoi tessellations in 2D, we used the voronoi and voronoi_plot_2d functions from the scipy.spatial module from SciPy (75), v1.11.1, available at: https://scipy.org/). Initial seeding positions were passed to the voronoi function to compute the tessellation, and voronoi_plot_2d was used for rendering the diagram. For 3D Voronoi diagrams, we employed a nearest-neighbor approximation method because standard packages do not directly support plotting. A regular 3D grid was created using numpy.meshgrid in NumPy (76) (v1.24.3, available at: https://numpy.org) and a set of seeding points representing colony centers was defined. scipy.spatial.KDTree (SciPy) was used to assign each grid point to its nearest seed, effectively partitioning the 3D space into Voronoi regions. For each region, the corresponding grid points were extracted and their convex hull was computed using scipy.spatial.ConvexHull (SciPy). Convex hulls were rendered as 3D polygons using Poly3DCollection from mpl_toolkits.mplot3d.art3d in Matplotlib (v3.7.1, available at https://matplotlib.org).

### Fluorescence Imaging of Biofilms.

Biofilms were imaged using a custom-built imager using a Lumencor SOLA SE II light engine (Lumencor, Beaverton, ORS), 474/27 and 575/25 excitation filters (Semrock, IDEX, West Henrietta, NY) and 525/45 and 641/75 emission filters (Semrock) for green and red fluorescence, respectively, a Canon EOS Rebel T5 DSLR containing an f/3.5 to 5.6 18 to 55 mm lens, and Canon Remote Capture software.

### Analysis of Image Similarity.

Binary masks defining regions of bacterial growth were created using the flood fill, morophology, and activecontour functions or the Segment Anything (77) tool in Matlab 2024b (Mathworks, Natick, MA). The similarity between experimental masks and masks created from RGM simulations was determined by computing the mean Chamfer distance (50), Jaccard index, and Pearson correlation coefficient. For fluorescence images, measurements were performed for each region containing a single fluorophore and weighted averages were determined for each metric using mask areas.

## Supplementary Material

Appendix 01 (PDF)

Movie S1. **Radial growth and Voronoi tessellation formation by *V. cholerae***. Timelapse images (left) of three V. cholerae populations inoculated in a uniform triangular arrangement on 0.2% agar over the course of 14 hours (same plates as shown in Fig. 1A). The Radial Growth Model (RGM) simulation (right) shows a similar expansion. Scale bar represents 1 cm.

Movie S2. **Voronoi tessellation during biofilm formation**. Timelapse images over the course of 88 hours of motility-defective V. cholerae strains (TW402 and TW421) on 0.05% methyl cellulose containing 0.2% hydroxyethyl agarose. The scale bar represents 1 cm.

Movie S3. **Voronoi tessellation by *Plesiomonas* and *Aeromonas***. Timelapse images (left) over the course of 14 hours of *Plesiomonas* (colony on left) and *Aeromonas* (colony on right) populations inoculated onto 0.2% agar (same plates as shown in Fig. 3C). RGM simulation (right) showing the expansion using the same initial arrangement but different expansion rates. Scale bar represents 1 cm.

## Data Availability

Raw data and scripts used for the Radial Growth Model data have been deposited in Zenodo (73) and Github (74), respectively. All other data are included in the manuscript and/or supporting information.
