# Supplementary material for: Geometric ordering in bacterial communities
Source: Proc Natl Acad Sci U S A. 2026 May 12;123(20):e2526643123. doi: 10.1073/pnas.2526643123 (PMC13187718; doi:10.1073/pnas.2526643123)
Supplement: Supplementary file 1 — Appendix 01 (PDF) [file pnas.2526643123.sapp.pdf]

PNAS

Supporting Information for

Geometric ordering in bacterial communities

Melika Gorgi, Summer J. Kasallis, Calvin Trinh, Lizett Ortiz de Ora, Travis Wiles, and Albert Siryaporn

Albert Siryaporn
Email: asirya@uci.edu

This PDF file includes:

Figures S1 to S2
Table S1
Legends for Movies S1 to S3

Other supporting materials for this manuscript include the following:

Movies S1 to S3

Figure S1

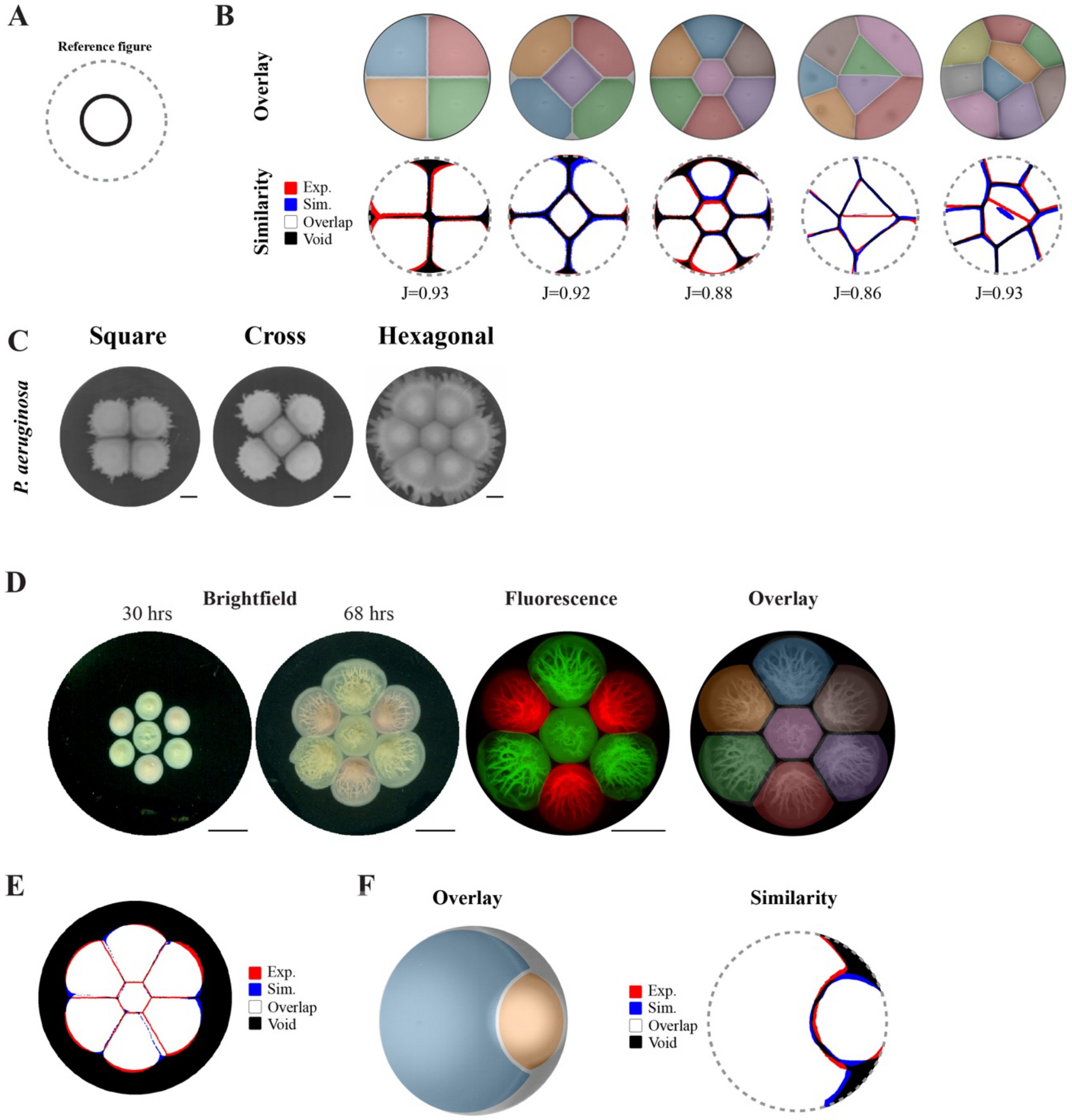

Figure S1. Analysis of Voronoi patterns formed by diverse bacterial species and conditions. (A) Image generated for reference for computing the Jaccard indices, Chamfer distance, and correlation coefficients (Table S1). **(B)** Grayscale brightfield images of *V. cholerae* in Fig. 2 overlaid with color RGM simulations (top) and comparison between experimental and simulation masks indicating overlap/agreement (white), where only the experiment (red) or simulation (blue) is observed, or where neither is observed (black) (bottom). **(C)** Images at 23 hours after inoculating *P. aeruginosa* in regular geometric arrangements (square, cross, and hexagonal) onto 0.5% agar. Images are of the same plates shown in Fig. 3A but at a later time point. **(D)** *V. cholerae* (TW402 and TW421) inoculated in a hexagonal configuration spaced 6 mm apart on media containing 0.15% methyl cellulose and 0.2% hydroxyethyl agarose, at the indicated incubation times. Fluorescence image at 68 hrs and the same image in grayscale with a hexagonal RGM simulation overlaid (right). **(E)** Comparison between experimental and RGM simulation for the hexagonal inoculation on liquid medium in Fig. 3B, indicating overlap/agreement (white), where only the experiment (red) or simulation (blue) is observed, or where neither is observed (black). **(F)** Overlay of color RGM simulation over grayscale image of *Plesiomonas* and *Aeromonas* growth in Fig. 3C at 13 hrs (left). Comparison between experimental mask and RGM simulation mask, indicating overlap/agreement (white), where only the experiment (red) or simulation (blue) is observed, or where neither is observed (black) (right). Scale bars represent 1 cm.

Figure S2

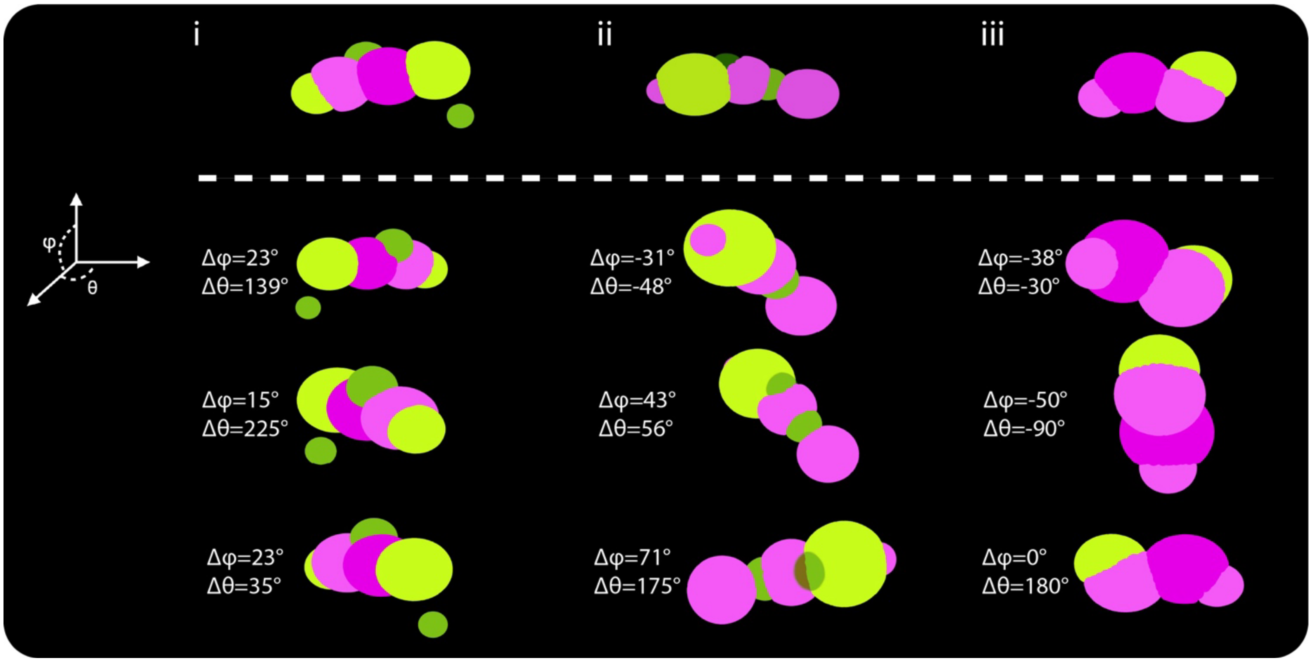

Figure S2. Multiple views of the 3D Radial Growth Model simulations for zebrafish colonization. The original simulation in Fig. 4 was rotated by the indicated polar (θ) and azimuthal (φ) angles to generate alternative views of the gut colonization.

Table S1

Bacterial Species	Environment	Inoculation geometry	Jaccard index J		Chamfer distance (mm)		Pearson correlation	
			Sim	Ref	Sim	Ref	Sim	Ref
V. cholerae	0.2% agar	Triangular	0.95	0.18	0.67	6.41	0.86	0.08
		Square	0.93	0.13	0.7	8.34	0.81	0.1
		Cross	0.92	0.13	0.66	6.58	0.72	0.06
		Hexagonal	0.88	0.13	0.66	7.04	0.7	0.1
		Disordered1	0.86	0.1	0.8	2.73	0.45	-0.1
		Disordered2	0.93	0.11	0.56	2.98	0.53	-0.06
P. aeruginosa	0.5% agar	Square	0.88	0.19	0.79	5.41	0.89	0.29
		Cross	0.85	0.15	0.85	6.12	0.86	0.20
		Hexagonal	0.86	0.15	0.66	6.73	0.83	0.23
V. cholerae	Methyl cellulose – hydroxyethyl agarose	Hexagonal	0.87	0.1	0.39	7.64	0.93	0.1
Plesiomonas & Aeromonas	0.2% agar	Linear	0.95	0.15	1.35	10.5	0.74	0.10
E. coli	Zebrafish gut i	Uncontrolled	0.77	0.08	0.0008	0.0102	0.86	0.03
	Zebrafish gut ii		0.75	0.1	0.0015	0.0142	0.82	0.06
	Zebrafish gut iii		0.74	0.02	0.0008	0.0098	0.84	-0.07

Table S1. Measurements of image similarity. Jaccard indices, Chamfer distances, and Pearson correlation coefficients between experimental and simulated images. A single image (Fig. S1A) was used as a reference for all samples.

Legends for Movies S1-S3

Movie S1. Radial growth and Voronoi tessellation formation by *V. cholerae*.
Timelapse images (left) of three *V. cholerae* populations inoculated in a uniform triangular arrangement on 0.2% agar over the course of 14 hours (same plates as shown in Fig. 1A). The Radial Growth Model (RGM) simulation (right) shows a similar expansion. Scale bar represents 1 cm.

Movie S2. Voronoi tessellation during biofilm formation.
Timelapse images over the course of 88 hours of motility-defective *V. cholerae* strains (TW402 and TW421) on 0.05% methyl cellulose containing 0.2% hydroxyethyl agarose. The scale bar represents 1 cm.

Movie S3. Voronoi tessellation by *Plesiomonas* and *Aeromonas*.
Timelapse images (left) over the course of 14 hours of *Plesiomonas* (colony on left) and *Aeromonas* (colony on right) populations inoculated onto 0.2% agar (same plates as shown in Fig. 3C). RGM simulation (right) showing the expansion using the same initial arrangement but different expansion rates. Scale bar represents 1 cm.
